# Occupational Health and Safety Practices Among Primary Healthcare Workers in Bangladesh: A Cross-Sectional Study

**DOI:** 10.7759/cureus.109963

**Published:** 2026-05-31

**Authors:** Aparna Mandal Anee, Sandipan Gain, Naima Ferdous, Nishat Jahan Nishi, Md Faizul Ahasan, Ahad Mahmud Khan, Md Shafiur Rahman

**Affiliations:** 1 Department of Occupational and Environmental Health, National Institute of Preventive and Social Medicine, Dhaka, BGD; 2 Department of Surgery, Faridpur General Hospital, Faridpur, BGD; 3 Department of Pharmacology and Therapeutics, Ibrahim Medical College and BIRDEM (Bangladesh Institute of Research and Rehabilitation in Diabetes, Endocrine and Metabolic Disorders) General Hospital, Dhaka, BGD; 4 Department of Public Health, Projahnmo Research Foundation, Dhaka, BGD

**Keywords:** bangladesh, healthcare workers, infection prevention, occupational health and safety, personal protective equipment, primary healthcare, workplace safety

## Abstract

Background

Healthcare workers (HCWs) in low- and middle-income countries are exposed to substantial occupational risks; however, evidence regarding comprehensive occupational health and safety (OHS) practices in primary healthcare settings remains limited. This study assessed OHS practices and explored factors associated with adequate OHS among primary HCWs in Bangladesh.

Methods

We conducted a facility-based cross-sectional study among 242 HCWs in eight sub-district hospitals in Bagerhat district, Bangladesh, in 2021. Data were collected using a structured questionnaire covering sociodemographic characteristics, infection prevention practices, personal protective equipment (PPE) use, working conditions, and occupational health services. A composite OHS practice index was constructed using eight binary indicators: handwashing before patient contact, handwashing after patient contact, handwashing for at least 30 seconds, mask use, glove use, goggle use, gown use, and receipt of PPE training. Working conditions, including rest during duty and meal availability, were assessed separately and were not included in the composite index. The total score ranged from 0 to 8, and a score of ≥5 was used as a predefined pragmatic threshold for meeting the composite criterion. Descriptive analysis and bivariable logistic regression were performed to explore associations with age, sex, profession, department, working hours, rest during duty, drinking water availability, training facilities, preventive medical check-up, and accident reporting system.

Results

Among the 242 participants, 190 were female (78.5%) and 174 were nurses (71.9%), indicating a demographically and professionally homogeneous sample. Hand hygiene before and after patient contact was reported by 214 participants (88.4%) and 238 participants (98.3%), respectively, although only 80 participants (33.1%) reported handwashing for at least 30 seconds. Mask use was reported by all participants (100.0%), while glove use was reported by 194 participants (80.2%). Only four participants (1.7%) reported using goggles and gowns, and 67 participants (27.7%) reported receiving PPE training. Less than half reported adequate rest during duty (47.1%), and none reported access to meals during duty. The mean OHS composite score was 4.31 ± 0.93. Using the predefined threshold of ≥5, 87 participants (36.0%) met the OHS composite criterion. No statistically significant associations were identified in bivariable analysis.

Conclusions

Only about one-third of HCWs met the predefined OHS composite criterion, highlighting important gaps in selected occupational safety measures, particularly PPE training, handwashing duration, and use of protective equipment beyond masks and gloves. The absence of significant predictors should be interpreted cautiously because the study was underpowered relative to the calculated sample size, used purposive sampling, and included a homogeneous sample dominated by female nurses. The findings should also be interpreted within the 2021 COVID-19 pandemic context, which may have influenced reported safety practices. Future studies should include larger and more occupationally diverse samples to better distinguish individual-level and institutional determinants of OHS practice in primary healthcare settings.

## Introduction

Occupational health and safety (OHS) is a fundamental component of resilient healthcare systems, ensuring the protection of healthcare workers (HCWs) from workplace hazards while maintaining the quality and continuity of patient care [1]. HCWs are routinely exposed to a wide range of occupational risks, including biological hazards such as infectious diseases, as well as physical, chemical, and psychosocial stressors. These risks are particularly pronounced in low- and middle-income countries (LMICs), where healthcare systems often operate under constraints related to limited resources, inadequate infrastructure, and insufficient enforcement of safety regulations [2].

Primary HCWs serve as the frontline of health systems in many LMICs, including Bangladesh, providing essential services such as outpatient care, maternal and child health services, and management of common infectious diseases. Frequent and close contact with patients increases their risk of occupational exposure, particularly in the absence of adequate infection prevention practices and personal protective equipment (PPE) [3]. The COVID-19 pandemic further highlighted vulnerabilities in healthcare systems, including gaps in PPE availability, insufficient training, and weak occupational health systems, which collectively contributed to increased risks among HCWs [4].

Standard infection prevention measures, such as proper hand hygiene and appropriate use of PPE, are well-established strategies to reduce occupational exposure [5]. However, adherence to these practices varies widely and is influenced by multiple factors, including availability of supplies, training, workload, and institutional support. In addition, workplace conditions, such as long working hours, insufficient rest, and lack of basic facilities, may affect both the ability and motivation of HCWs to adhere to recommended safety practices [6]. Despite these considerations, evidence on comprehensive OHS practices in primary healthcare settings in LMICs remains limited, particularly when multiple domains of safety are assessed simultaneously [7].

In Bangladesh, existing studies have primarily focused on selected aspects of infection prevention and control, often within hospital settings, with limited attention to primary healthcare facilities. Moreover, many studies rely on single indicators or descriptive measures, which may not adequately capture the multidimensional nature of OHS. Composite indices that integrate multiple practices offer a more comprehensive assessment but require careful consideration of how adequacy is defined, as overly lenient thresholds may overestimate true performance [8,9].

Therefore, this study aimed to assess OHS practices among HCWs in subdistrict-level primary healthcare facilities in Bangladesh using a composite index and to explore factors associated with meeting a predefined OHS composite criterion. The composite index focused on infection prevention and PPE-related domains, while working conditions and institutional factors were assessed separately. Because no validated cutoff was available for this setting, a score of ≥5 out of 8 was used as a pragmatic, predefined threshold, representing adherence to more than half of the selected OHS indicators. The findings are intended to provide an exploratory assessment of key safety gaps and inform future research and interventions to improve occupational safety in primary healthcare settings.

## Materials and methods

Study design and setting

This facility-based cross-sectional study was conducted between January and December 2021 in eight sub-district hospitals in the Bagerhat district, Bangladesh. These facilities represent primary healthcare settings and provide essential services, including outpatient care, inpatient care, maternal and child health services, laboratory services, and management of common infectious diseases. The study period coincided with the COVID-19 pandemic, which may have influenced infection prevention practices, PPE use, and institutional safety procedures.

Study population and sampling

The study population included HCWs directly involved in patient care, including physicians, nurses, and medical technologists. Eligible participants were those working in the selected facilities during the study period and available at the time of data collection. HCWs who were absent during facility visits, not involved in direct patient care, or unwilling to provide written informed consent were excluded. A purposive sampling approach was used because the study was conducted under operational constraints during the COVID-19 pandemic. Participants were approached at their workplaces during facility visits by trained data collectors. The study team explained the purpose of the study, obtained written informed consent, and conducted face-to-face interviews in a private or semi-private area within the facility whenever feasible. A total of 242 HCWs were enrolled. As the total number of eligible HCWs and the number approached were not systematically recorded across all facilities, a formal response rate could not be calculated.

Sample size calculation

The sample size was calculated using the single population proportion formula: n = Z²p(1-p)/d². As no prior estimate of comprehensive OHS practice among primary HCWs in the selected setting was available, a conservative expected proportion of 50% was used to obtain the maximum sample size. Using a 95% confidence level, 5% margin of error, and a Z value of 1.96, the minimum required sample size was 384. However, due to operational constraints during the COVID-19 pandemic, purposive facility-based recruitment, and the finite number of eligible HCWs available during data collection visits, 242 HCWs were enrolled. The smaller-than-calculated sample size may have reduced statistical power, particularly for detecting factors associated with the composite OHS outcome.

Data collection tools and procedures

Data were collected through face-to-face interviews using a pretested structured questionnaire (see Appendix) developed based on established OHS frameworks and relevant literature [10-13]. The questionnaire collected information on sociodemographic characteristics, including age, sex, profession, and department. It also assessed infection prevention practices, including hand hygiene and PPE use; working conditions, including working hours, rest during duty, and availability of drinking water and meals; and occupational health services, including training, preventive medical check-ups, and accident reporting systems.

Questionnaire development and validation

The initial questionnaire was reviewed by public health and occupational health experts to assess content relevance, clarity, and appropriateness for the primary healthcare setting. The tool was then pretested among HCWs in a comparable healthcare setting who were not included in the final analysis. Based on expert feedback and pretest findings, the wording and sequencing of selected questions were revised to improve clarity and flow. The questionnaire was administered in Bangla by trained data collectors. No formal reliability testing, such as Cronbach’s alpha or factor analysis, was conducted because the composite index was developed for this exploratory study.

Outcome variable: OHS practice composite index

The primary outcome was meeting the predefined OHS practice composite criterion. The composite index was derived from eight binary indicators across infection prevention, PPE use, and PPE training domains: handwashing before patient contact, handwashing after patient contact, handwashing for a duration of at least 30 seconds, mask use, glove use, goggle use, gown use, and receipt of PPE training. Each indicator was coded as 1 if the recommended practice or enabling condition was reported and 0 otherwise, resulting in a total score ranging from 0 to 8. Although receipt of PPE training is not a direct behavioral practice, it was included in the composite index as an enabling component of OHS readiness. All indicators were weighted equally for pragmatic reasons and to provide a simple exploratory summary of selected OHS-related domains. A score of ≥5 was used as a predefined pragmatic threshold for meeting the OHS composite criterion. This threshold was selected because it represented adherence to more than half of the selected indicators and provided greater discrimination than a median-based cutoff in this dataset. However, this cutoff should not be interpreted as a validated clinical standard for adequate OHS practice. Working conditions and occupational health service indicators, including rest during duty, meal availability, drinking water availability, availability of training facilities, preventive medical check-ups, and accident reporting systems, were not included in the composite index and were analyzed separately. Details of the indicators and scoring approach are provided in the Appendix.

Independent variables

Independent variables included sociodemographic characteristics (age, sex, profession, and department), workplace factors (working hours, rest during duty, and availability of drinking water), and institutional factors (training facilities, preventive medical check-ups, and accident reporting systems).

Data transformation and missing data

Responses from the questionnaire were recoded into binary variables for the construction of the OHS composite index. For handwashing duration, responses indicating at least 30 seconds were coded as 1, while responses below 30 seconds were coded as 0. For PPE indicators, “yes” responses were coded as 1 and “no” responses were coded as 0. The ≥30-second threshold was used based on the available questionnaire response categories. Missing data were checked during data cleaning. Participants with complete information on the eight OHS composite indicators were included in the composite score analysis.

Statistical analysis

Data were entered, cleaned, and analyzed using the Statistical Package for the Social Sciences (SPSS) version 26 (IBM Inc., Armonk, NY). Descriptive statistics were used to summarize participant characteristics, working conditions, occupational health services, and individual OHS indicators. Frequencies and percentages were reported for categorical variables. The distribution of the composite OHS score was summarized using the mean, standard deviation (SD), median, and interquartile range. Bivariate logistic regression analysis was performed to explore associations between participant and workplace characteristics and meeting the predefined OHS composite criterion. Variables assessed included age, sex, profession, department, working hours, rest during duty, drinking water availability, availability of training facilities, preventive medical check-ups, and accident reporting systems. Crude odds ratios with 95% confidence intervals (CIs) were reported. Because only one variable met the predefined p < 0.20 threshold for multivariable model entry, a multivariable model was not presented. This decision was made to avoid presenting a single-variable model as an adjusted analysis. The potential clustering of participants within facilities was not accounted for analytically because of the small number of facilities and the exploratory nature of the analysis. All statistical tests were two-sided, and statistical significance was set at p < 0.05.

Ethical considerations

Ethical approval was obtained from the Institutional Review Board of the National Institute of Preventive and Social Medicine (NIPSOM), Dhaka, Bangladesh (Memo No.: NIPSOM/IRB/2021/18). Written informed consent was obtained from all participants prior to data collection. The confidentiality of participants’ information was maintained throughout the study.

## Results

Sociodemographic and work characteristics

Table 1 presents the sociodemographic and basic characteristics of the participants. Among the 242 HCWs, the majority were aged 24-38 years (69.0%). Most participants were female (78.5%). In terms of profession, 71.9% were nurses, 19.8% were doctors, and 8.3% were medical technologists. Participants were distributed across outpatient, inpatient/ward, laboratory, and other departments.

**Table 1:** Sociodemographic and work characteristics of healthcare workers (N = 242)

Variable	n (%)
*Age group (years)*	
24–38	167 (69.0)
39–53	61 (25.2)
>53	14 (5.8)
*Sex*	
Male	52 (21.5)
Female	190 (78.5)
*Profession*	
Doctor	48 (19.8)
Nurse	174 (71.9)
Medical technologist	20 (8.3)
*Department*	
Inpatient/Ward	117 (48.3)
Outpatient	107 (44.2)
Laboratory	9 (3.7)
Others	9 (3.7)

Occupational health and safety practices

Table 2 presents OHS practices among HCWs. Most of them reported practicing hand hygiene before (88.4%) and after (98.3%) patient contact; however, only 33.1% adhered to the recommended handwashing duration of at least 30 seconds. Mask use was universal (100.0%), while glove use was reported by 80.2% of participants. In contrast, the use of goggles and gowns was very low (1.7% each). Only 27.7% of HCWs reported receiving formal training on PPE use.

**Table 2:** Occupational health and safety practices (N = 242) PPE: Personal protective equipment.

Domain/Indicator	Yes, n (%)
Infection prevention	
Handwashing before patient contact	214 (88.4)
Handwashing after patient contact	238 (98.3)
Handwashing ≥30 seconds	80 (33.1)
PPE use	
Mask use	242 (100.0)
Glove use	194 (80.2)
Goggle use	4 (1.7)
Gown use	4 (1.7)
Training	
PPE training received	67 (27.7)

Working conditions and occupational health services

Table 3 presents the working conditions and availability of occupational health services. Most participants worked six days per week (98.6%), and about 20% worked more than eight hours per day. Less than half of the participants reported having adequate rest during duty (47.1%), and none reported access to meals during working hours. Drinking water was available to 43.4% of HCWs during duty. Although 91.3% of participants reported the availability of training facilities at their institutions, only 27.7% reported receiving formal PPE training. This gap between institutional availability of training facilities and actual receipt of PPE training indicates an important operational weakness in translating available resources into staff-level occupational safety preparedness.

**Table 3:** Working conditions and occupational health services (N = 242)

Variable	Yes, n (%)
*Working conditions*	
Working 6 days per week	239 (98.8)
Working ≤ 8 hours per day	195 (80.6)
Adequate rest during duty	114 (47.1)
Meal availability during duty	0 (0.0)
Drinking water availability	105 (43.4)
*Occupational health services*	
Training facilities available	221 (91.3)
Preventive medical check-up	144 (59.5)
Accident reporting system	99 (40.9)
Health surveillance system	0 (0.0)

Distribution of the OHS composite score and classification

The composite OHS score ranged from 1 to 7, with a mean (SD) of 4.31 (0.93) and a median (IQR) of 4 (4-5). Using a more stringent threshold of ≥5, 87 participants (36.0%) were classified as having adequate OHS practices, while 155 (64.0%) were classified as having inadequate OHS practices.

Factors associated with meeting the predefined OHS composite criterion

Bivariate logistic regression analysis was conducted to explore factors associated with meeting the predefined OHS composite criterion (Table 4). Variables assessed included age, sex, profession, working hours, rest during duty, drinking water availability, training facilities, preventive medical check-ups, and accident reporting systems. None of the examined variables were statistically significantly associated with meeting the OHS composite criterion at the 5% level.

**Table 4:** Bivariable logistic regression analysis of factors associated with adequate OHS practices among healthcare workers OHS: Occupational health and safety; COR: Crude odds ratio; CI: Confidence interval.

Variable	Adequate n (%)	Inadequate n (%)	COR (95% CI)	p-value
*Age group (years)*				
24–38	61 (36.5)	106 (63.5)	Reference	—
39–53	20 (32.8)	41 (67.2)	0.85 (0.46–1.58)	0.602
>53	6 (42.9)	8 (57.1)	1.30 (0.43–3.93)	0.638
*Sex*				
Male	17 (32.7)	35 (67.3)	Reference	—
Female	70 (36.8)	120 (63.2)	1.34 (0.70–2.59)	0.381
*Profession*				
Doctor	16 (33.3)	32 (66.7)	Reference	—
Nurse	65 (37.4)	109 (62.6)	1.19 (0.61–2.34)	0.608
Medical technologist	6 (30.0)	14 (70.0)	0.86 (0.28–2.65)	0.789
*Rest during duty*				
No	52 (40.6)	76 (59.4)	Reference	—
Yes	35 (30.7)	79 (69.3)	0.65 (0.38–1.10)	0.109
*Drinking water availability*				
No	43 (31.4)	94 (68.6)	Reference	—
Yes	44 (41.9)	61 (58.1)	1.36 (0.80–2.31)	0.251
*Training facilities*				
No	6 (28.6)	15 (71.4)	Reference	—
Yes	81 (36.7)	140 (63.3)	1.45 (0.54–3.88)	0.463
*Medical check-up*				
No	30 (30.6)	68 (69.4)	Reference	—
Yes	57 (39.6)	87 (60.4)	1.38 (0.80–2.36)	0.249
*Accident reporting*				
No	55 (38.5)	88 (61.5)	Reference	—
Yes	32 (32.3)	67 (67.7)	0.82 (0.48–1.41)	0.480
*Working ≤ 8 hours/day*				
No (>8 hours)	15 (31.9)	32 (68.1)	Reference	—
Yes	72 (36.9)	123 (63.1)	1.41 (0.71–2.81)	0.328
Total	87 (36.0)	155 (64.0)		

HCWs reporting adequate rest during duty had lower odds of meeting the OHS composite criterion compared with those without adequate rest, although this was not statistically significant. This non-significant trend should be interpreted cautiously and may reflect differences in work setting, workload, or infection prevention enforcement rather than a direct effect of rest on OHS behavior. As only one variable met the predefined criterion for inclusion in a multivariable model, adjusted regression analysis was not performed.

## Discussion

This study assessed OHS practice among HCWs in subdistrict-level primary healthcare facilities in Bangladesh using a composite index of selected infection prevention, PPE use, and PPE training indicators. Only 36.0% of participants met the predefined OHS composite criterion, indicating substantial gaps in comprehensive occupational safety measures in this sample. This proportion should be interpreted as the percentage meeting the study-specific composite threshold, rather than as a definitive estimate of adequate OHS practice based on an externally validated standard.

Although basic infection prevention practices were widely reported, important deficiencies were observed in key components of comprehensive OHS. Nearly all participants reported handwashing after patient contact, and mask use was universal. However, only one-third adhered to the questionnaire-defined handwashing duration of at least 30 seconds. Use of goggles and gowns was extremely low, and fewer than one-third of HCWs reported formal PPE training. The large discrepancy between reported availability of institutional training facilities and actual receipt of PPE training suggests that the presence of training infrastructure alone may not ensure effective staff coverage or practical preparedness. These findings suggest that while certain routine practices are well-established, comprehensive protection, particularly for exposure to droplets and bodily fluids, remains inadequate. Similar gaps between basic compliance and full adherence to infection prevention standards have been reported in other low-resource settings [2,5,14-18].

The study was conducted in 2021, during the COVID-19 pandemic, which is important for interpreting the findings. Universal mask use likely reflects pandemic-related mandates and heightened awareness rather than routine pre-pandemic OHS practice. Conversely, the very low use of goggles and gowns may reflect limited supply, prioritization of selected PPE, or use only in specific exposure scenarios. Therefore, these findings should be understood as reflecting a pandemic-era primary healthcare context and may not fully represent non-crisis or post-pandemic OHS conditions.

The study also highlights the importance of how OHS is measured. When a median-based cutoff was applied, a large majority of participants were classified as having adequate OHS practices; however, this threshold was found to overestimate adequacy due to the skewed distribution of the composite score and the high prevalence of certain indicators, such as mask use. By applying a more stringent threshold (≥5), the proportion of adequate OHS practices decreased substantially, providing a more realistic assessment. This underscores the need for careful consideration in constructing and interpreting composite indices, particularly in contexts where some practices are nearly universal.

No statistically significant predictors of meeting the OHS composite criterion were identified. However, this should not be interpreted as evidence that individual-level factors are unimportant. The null findings may be explained by the smaller-than-calculated sample size, purposive sampling, limited variability in key predictors, and the demographic and professional homogeneity of the sample, which was predominantly female and composed largely of nurses. These factors may have reduced statistical power and increased the likelihood of a type II error.

The non-significant negative association between adequate rest during duty and meeting the OHS composite criterion was counterintuitive. One possible explanation is that HCWs in higher workload areas may have experienced stricter infection prevention supervision or greater perceived infection risk, leading to higher reported PPE and hand hygiene practices despite less rest. Conversely, workers in lower workload settings may have had more opportunities for rest but less frequent exposure to high-risk situations or less consistent enforcement of PPE practices. As this association was not statistically significant and the study was cross-sectional, it should be considered exploratory.

The absence of significant predictors may reflect several factors. First, OHS practices may be influenced by broader systemic or institutional determinants that were not fully captured in this study, such as supervision, organizational culture, availability and consistency of supplies, and enforcement of guidelines. Second, limited variability in some independent variables, such as high reported availability of training facilities, may have reduced the ability to detect meaningful associations. Third, the composite outcome itself may have limited discriminatory power, particularly when combining indicators with varying levels of importance and prevalence [19-21].

This study also has several limitations. First, the cross-sectional design precludes causal inference. Second, OHS practices were self-reported and may be affected by recall and social desirability bias. Third, the study enrolled 242 participants rather than the calculated minimum sample size of 384, which reduced statistical power, particularly for regression analysis. Fourth, purposive sampling based on participant availability may have introduced selection bias, and the findings may not be representative of all primary HCWs in Bangladesh. Fifth, the sample was demographically and professionally homogeneous, with most participants being female and nurses, limiting variability for identifying predictors of OHS practice. Sixth, the OHS composite index was developed for this exploratory study and has not been externally validated. The index combined heterogeneous indicators, including routine behaviors, task-dependent PPE use, and receipt of PPE training, which may not represent a single underlying construct. Equal weighting of all indicators may not reflect their relative importance or clinical relevance. In addition, goggles and gowns may not be required for every patient encounter; therefore, scoring non-use as inadequate without assessing clinical indication may underestimate appropriate PPE adherence. Seventh, the ≥5 threshold was a pragmatic predefined criterion and should not be interpreted as a validated clinical benchmark. Eighth, the study was conducted during the COVID-19 pandemic, which may have influenced mask use, PPE availability, training exposure, and institutional safety practices. Finally, participants were nested within eight facilities, but facility-level clustering was not accounted for analytically, which may have affected precision estimates.

The findings of this study have important implications for policy and practice. The low prevalence of adequate OHS practices indicates the need for strengthening comprehensive infection prevention and occupational safety measures in primary healthcare settings. Improving access to and utilization of PPE beyond masks and gloves, ensuring regular and practical training on PPE use, and strengthening institutional systems such as reporting mechanisms and supervision are critical steps. In addition, attention should be given to ensuring adequate working conditions, including rest and basic facilities, which may indirectly influence adherence to safety practices [22-24].

## Conclusions

This study found important gaps in OHS practice among primary HCWs in selected subdistrict-level facilities in Bangladesh. While selected infection prevention practices, particularly mask use and handwashing after patient contact, were widely reported, comprehensive protection was limited by low PPE training coverage, inadequate handwashing duration, and very low reported use of goggles and gowns. Only 36.0% of participants met the predefined OHS composite criterion. The absence of statistically significant predictors should be interpreted with caution, as individual-level associations cannot be ruled out given the smaller-than-calculated sample size, purposive sampling, and limited variability in the study population. The findings suggest the need to strengthen sustainable occupational safety systems in primary healthcare settings, including regular practical PPE training, supportive supervision, a reliable supply of appropriate PPE, clear reporting mechanisms, and institutional policies that promote a long-term safety culture beyond crisis-period responses.

## Appendices

**Table 5:** Operational definition and scoring of occupational health and safety practice index Each indicator was coded as 1 = practice present/yes and 0 = practice absent/no. The composite OHS practice index was calculated by summing the eight indicators, giving a possible score range of 0–8. Respondents scoring ≥5 were categorized as having adequate OHS practice, while those scoring <5 were categorized as having inadequate OHS practice. This practice index focuses on infection prevention and PPE-related OHS practices available in the dataset. OHS: Occupational health and safety; PPE: Personal protective equipment.

Domain	Indicator	Operational definition for score = 1	Score
Infection prevention practices	Hand washing before patient contact	Respondent reported performing hand washing before patient contact	0–1
	Hand washing after patient contact	Respondent reported performing hand washing after patient contact	0–1
	Adequate hand-washing duration	Respondent reported washing hands for ≥30 seconds	0–1
Personal protective equipment (PPE) practices/training	Mask use	Respondent reported using a mask during patient care	0–1
	Glove use	Respondent reported using gloves during patient care	0–1
	Goggle use	Respondent reported using goggles during patient care	0–1
	Gown use	Respondent reported using a gown during patient care	0–1
	PPE training received	Respondent reported receiving formal training on PPE use	0–1
Total OHS practice score	—	Sum of all eight binary indicators	0–8
Classification	Adequate OHS practice	Total score ≥ 5, equivalent to achieving at least 60% of the maximum score	—
	Inadequate OHS practice	Total score < 5	—

**Table 6:** Study questionnaire

S. No.	Variable/Question	Response Options/Coding
Sociodemographic information		
1	Age in years	____________
2	Gender	Male = 1, Female = 2
3	Religion	Muslim = 1, Hindu = 2, Buddhism = 3, Christianity = 4
4	Marital status	Unmarried = 1, Married = 2, Divorced = 3, Widow = 4, Separated = 5
5	Professional category of healthcare worker	Doctor = 1, Nurse = 2, Lab/Medical technologist = 3
6	Working department of healthcare workers	Emergency department = 1, Outdoor = 2, Indoor = 3, Laboratory = 4, Others = 5
Occupational risk-related information		
7	Do you provide direct service to patients with infectious diseases in the Upazila Health Complex?	Yes = 1, No = 0
8	Do you wash your hands before patient contact in the health complex?	Yes = 1, No = 0
9	If yes, do you use soap and water to cleanse your hands?	Yes = 1, No = 0
10	If yes, do you use only water to cleanse your hands?	Yes = 1, No = 0
11	If yes, do you use alcohol-based sanitizer to cleanse your hands?	Yes = 1, No = 0
12	Do you wash your hands after patient contact in the health complex?	Yes = 1, No = 0
13	If yes, do you use soap and water to cleanse your hands?	Yes = 1, No = 0
14	If yes, do you use only water to cleanse your hands?	Yes = 1, No = 0
15	If yes, do you use alcohol-based sanitizer to cleanse your hands?	Yes = 1, No = 0
16	Duration of hand washing	Less than 30 seconds = 1, 30 seconds or more = 2
17	How many times do you cleanse your hands per day?	____________
18	Do you use PPE during patient care?	Yes = 1, No = 0
19	If yes, do you use gloves?	Yes = 1, No = 0
20	If yes, do you use a mask?	Yes = 1, No = 0
21	If yes, do you use goggles?	Yes = 1, No = 0
22	If yes, do you use a gown?	Yes = 1, No = 0
23	Do you have facilities for donning and doffing of PPE in your health complex?	Yes = 1, No = 0
24	Do you have any training on the use of PPE?	Yes = 1, No = 0
25	Is there a room for chemical/disinfectant storage locally?	Yes = 1, No = 0
26	Is there a room for chemical/disinfectant disposal locally?	Yes = 1, No = 0
Occupational environment-related information		
27	How many days do you work in a week?	____________
28	How many hours do you work in a week?	____________
29	How many hours do you work every day?	____________
30	Do you have sufficient time for rest?	Yes = 1, No = 0
31	Do you have any duty meal service?	Yes = 1, No = 0
32	Do you have facilities for the supply of drinking water during duty?	Yes = 1, No = 0
Occupational healthcare-related information		
33	Have you received any training in your health complex?	Yes = 1, No = 0
34	Do you have vaccination facilities in this health complex?	Yes = 1, No = 0
35	Do you have preventive health check-up facilities in this health complex?	Yes = 1, No = 0
36	Do you have facilities for the collection of health surveillance-related information in your health complex?	Yes = 1, No = 0
37	Do you have reporting facilities for occupational accidents in your health complex?	Yes = 1, No = 0

## References

[1] Huei LC, Ya-Wen L, Ming YC, Chen HL, Yi WJ, Hung LM (2020). Occupational health and safety hazards faced by healthcare professionals in Taiwan: a systematic review of risk factors and control strategies. SAGE Open Med.

[2] Rai R, El-Zaemey S, Dorji N, Rai BD, Fritschi L (2021). Exposure to occupational hazards among health care workers in low- and middle-income countries: a scoping review. Int J Environ Res Public Health.

[3] Mawuena EK, Mannion R (2022). Implications of resource constraints and high workload on speaking up about threats to patient safety: a qualitative study of surgical teams in Ghana. BMJ Qual Saf.

[4] Cohen J, Rodgers YV (2020). Contributing factors to personal protective equipment shortages during the COVID-19 pandemic. Prev Med.

[5] Alhumaid S, Al Mutair A, Al Alawi Z (2021). Knowledge of infection prevention and control among healthcare workers and factors influencing compliance: a systematic review. Antimicrob Resist Infect Control.

[6] Babore GO, Eyesu Y, Mengistu D, Foga S, Heliso AZ, Ashine TM (2024). Adherence to infection prevention practice standard protocol and associated factors among healthcare workers. Glob J Qual Saf Healthc.

[7] Langlois EV, McKenzie A, Schneider H, Mecaskey JW (2020). Measures to strengthen primary health-care systems in low- and middle-income countries. Bull World Health Organ.

[8] Biswas MAJ, Hassan MZ, Monjur MR (2021). Assessment of standard precaution related to infection prevention readiness of healthcare facilities in Bangladesh: findings from a national cross-sectional survey. Antimicrob Steward Healthc Epidemiol.

[9] Harun MG, Anwar MM, Sumon SA (2022). Infection prevention and control in tertiary care hospitals of Bangladesh: results from WHO infection prevention and control assessment framework (IPCAF). Antimicrob Resist Infect Control.

[10] (2026). Occupational health: health workers. https://www.who.int/news-room/fact-sheets/detail/occupational-health--health-workers.

[11] (2026). Safety and health at work. https://www.ilo.org/topics-and-sectors/safety-and-health-work.

[12] (2026). WHO-ILO Global Framework for National Occupational Health Programmes for Health Workers. https://www.who.int/news/item/10-06-2020-who-ilo-global-framework-for-national-occupational-health-programmes-for-health-workers.

[13] (2026). Caring for those who care: guide for the development and implementation of occupational health and safety programmes for health workers. https://www.who.int/publications/i/item/9789240040779.

[14] Odonkor ST, Sallar AM (2024). Occupational health and safety knowledge, attitudes and practices among healthcare workers in Accra Ghana. Scientific African.

[15] Dhahir DM, Al Mayahi NY (2021). Assessment of health workers knowledge toward occupational health and safety program in Alkut city’s primary health care centers. Medico Legal Update.

[16] Yilma M, Taye G, Abebe W (2024). Magnitude of standard precautions practices among healthcare workers in health facilities of low and middle income countries: a systematic review and meta-analysis. PLoS One.

[17] Akagbo SE, Nortey P, Ackumey MM (2017). Knowledge of standard precautions and barriers to compliance among healthcare workers in the Lower Manya Krobo District, Ghana. BMC Res Notes.

[18] Reda AA, Fisseha S, Mengistie B, Vandeweerd JM (2010). Standard precautions: occupational exposure and behavior of health care workers in Ethiopia. PLoS One.

[19] Debela MB, Deyessa N, Begosew AM, Azage M (2023). Occupational health and safety practices and associated factors among workers in Ethiopia's Metehara and Wonji sugar industries: a convergent parallel mixed design. BMJ Open.

[20] Hussein A, Opoku-Mensah FA, Amoafo IQ, Saifu K (2025). Fundamentals of occupational health and safety practices outside the workplace among health professionals in Ghana: an assessment of determinants of crash helmet use in the Tamale Teaching Hospital. BMC Public Health.

[21] Zhou S, Chen H, Wu J, Xia B, Yu X, Huang M, Yang M (2025). Associations of socioeconomic and occupational characteristics with occupational health literacy among essential service workers in Guangdong, China. BMC Public Health.

[22] George J, Shafqat N, Verma R, Patidar AB (2023). Factors influencing compliance with personal protective equipment (PPE) use among healthcare workers. Cureus.

[23] Mohammad A (2023). Recognizing and preventing occupational health hazards: a critical need for a competent and trained healthcare workforce and a robust public health surveillance system. J Dow Univ Health Sci.

[24] Janjua NZ, Razaq M, Chandir S, Rozi S, Mahmood B (2007). Poor knowledge--predictor of nonadherence to universal precautions for blood borne pathogens at first level care facilities in Pakistan. BMC Infect Dis.

